# Sales of Iodine-Containing Drugs in Europe Following the Beginning of the War Between Russia and Ukraine

**DOI:** 10.1001/jamanetworkopen.2022.40032

**Published:** 2022-10-27

**Authors:** Karel Kostev, Susanne Abeler, Ai Koyanagi, Josep Maria Haro, Lee Smith, Louis Jacob

**Affiliations:** 1 Epidemiology, IQVIA, Frankfurt, Germany; 2 Consumer Health, IQVIA, Frankfurt, Germany; 3 Research and Development Unit, Parc Sanitari Sant Joan de Déu, Centro de Investigación Biomédica en Red de Salud Mental, Instituto de Salud Carlos III, Barcelona, Spain; 4 Institució Catalana de Recerca i Estudis Avançats, Barcelona, Spain; 5 Centre for Health, Performance, and Wellbeing, Anglia Ruskin University, Cambridge, United Kingdom; 6 Faculty of Medicine, University of Versailles Saint-Quentin-en-Yvelines, Montigny-le-Bretonneux, France

## Abstract

This cross-sectional study investigates sales of over-the-counter iodine-containing drugs in 20 European countries between January 2021 and March 2022.

## Introduction

In February 2022, Russia invaded Ukraine and aimed to control the country rapidly.^1,2^ The Russia-Ukraine war has raised multiple concerns globally. Two major concerns are the potential use of nuclear weapons and the environmental consequences of the capture of nuclear sites by the Russian army.^1^ A nuclear war would result in many immediate deaths and delayed deaths from chronic diseases and climate disruption. Although guidelines from the World Health Organization advocate the prudent prophylactic use of iodine,^3^ media outlets have reported panic buying of iodine-containing drugs in Europe and other regions of the world since the beginning of the Russia-Ukraine conflict.^4^ Given that, to our knowledge, there is no research on this topic yet, the present pharmacoepidemiological study aimed to investigate sales of over-the-counter iodine-containing drugs in 20 European countries between January 2021 and March 2022.

## Methods

This cross-sectional study used data from the OTCims database (IQVIA), which contains data on sales of over-the-counter drugs in Europe. The present study included sell-out data of drugs with iodine as the only active ingredient. The number of packages of iodine-containing drugs sold per month was analyzed in each country between January 2021 and March 2022. Differences in percentage were assessed between February 2022 and January 2022, and March 2022 and January 2022 (eAppendix in the Supplement). The database used includes only anonymized data in compliance with the regulations of the applicable data protection laws. German law allows the use of anonymous data for research purposes under certain conditions. According to this legislation, it is not necessary to obtain approval from a medical ethics committee for this type of observational study that contains no directly identifiable data. Informed consent was not needed because no patient data were used, in accordance with German laws.

## Results

Channels of distribution, sample size, and coverage by country are shown in Table 1. In January 2021, the number of packages of iodine-containing drugs sold was the highest in Russia (405 437 packages) and the lowest in Portugal (7 packages). The number of sales of packages of iodine-containing drugs increased in most countries in February 2022 and all countries in March 2022 compared with January 2022. This increase was particularly strong in Romania (29 339.3% in March 2022), Bulgaria (14 507.3% in March 2022), and the Netherlands (8765.7% in March 2022) (Table 2).

**Table 1.** Channels of Distribution, Sample Size, and Coverage by Country

Country	Channels of distribution	Pharmacies in the country, No.	Pharmacies in the database, No. (%)	Coverage
Austria	Retail pharmacies	1390	495 (35.6)	Projected
Belgium	Retail pharmacies	4850	2500 (51.5)	Projected
Bulgaria	Retail pharmacies, online and mail orders	3049	670 (22.0)	Not projected
Croatia	Retail pharmacies, online and mail orders	1148	302 (26.3)	Projected
Czech Republic	Retail pharmacies, online and mail orders	2800	1350 (48.2)	Projected
Finland	Retail pharmacies	810	Not available	Not available
France	Retail pharmacies	21 242	14 000 (65.9)	Projected
Germany	Retail pharmacies, drugstores, supermarket aisles and shelves, online and mail orders	19 205	Not available (19.0)	Projected
Greece	Retail pharmacies	10 100	2050 (20.3)	Projected
Hungary	Retail pharmacies	2373	730 (30.8)	Projected
Italy	Retail pharmacies, supermarket in-store pharmacies and corners, supermarket aisles and shelves, other channels	19 267	8351 (43.3)	Projected
Latvia	Retail pharmacies, online and mail orders, other channels	789	450 (57.0)	Projected
Netherlands	Retail pharmacies, drugstores, supermarket in-store pharmacies and corners, supermarket aisles and shelves	2021	925 (45.8)	Projected
Poland	Retail pharmacies, online and mail orders	13 752	6500 (47.3)	Projected
Portugal	Retail pharmacies, supermarket in-store pharmacies and corners, other channels	2905	1600 (55.1)	Projected
Romania	Retail pharmacies, supermarket in-store pharmacies and corners	7366	3071 (41.7)	Not available
Russia	Retail pharmacies, supermarket in-store pharmacies and corners	51 500	8300 (16.1)	Projected
Slovakia	Retail pharmacies, online and mail orders	1960	720 (36.7)	Projected
Spain	Retail pharmacies	22 000	6300 (28.6)	Projected
Switzerland	Retail pharmacies, drugstores	1787	973 (54.4)	Projected

**Table 2.** Number of Packages of Iodine-Containing Drugs Sold Each Month in 20 European Countries Between January 2021 and March 2022

Country	Absolute No. of packages, by year and month															Feb 2022 vs Jan 2022 increase, %	Mar 2022 vs Jan 2022 increase, %
	2021												2022				
	Jan	Feb	Mar	Apr	May	Jun	Jul	Aug	Sep	Oct	Nov	Dec	Jan	Feb	Mar		
Austria	150	151	161	248	294	192	201	135	159	225	223	151	230	2393	9418	940.4	3994.8
Belgium	575	511	583	530	680	628	581	500	503	569	435	472	476	674	891	41.6	87.2
Bulgaria	38	75	45	5	56	45	18	58	58	8	83	10	41	103	5989	151.2	14 507.3
Croatia	75	49	59	86	107	92	97	64	97	155	110	114	71	175	225	146.5	216.9
Czech Republic	3817	3556	4174	4159	4350	4507	3445	3247	3120	3117	3417	3248	3375	8551	15 029	153.4	345.3
Finland	613	536	897	767	619	584	393	601	576	784	628	605	1098	9594	93 869	773.8	8449.1
France	4641	4437	4836	4751	4430	4888	4686	4239	4779	4701	4812	4748	5032	6821	16 997	35.6	237.8
Germany	173 504	157 053	183 232	171 641	165 286	170 115	180 003	170 220	165 774	174 339	181 944	174 347	165 575	254 411	497 861	53.7	200.7
Greece	57	40	41	56	58	8	18	34	20	33	38	11	63	38	317	−39.7	403.2
Hungary	1622	1699	1757	1551	1687	1734	1707	1599	1622	1750	1782	1575	1605	4361	14 846	171.7	825.0
Italy	5611	5376	6467	6233	5899	5771	5941	5051	5926	5693	5487	5959	5646	5790	20 588	2.6	264.6
Latvia	33	16	36	27	38	25	21	13	16	32	15	15	17	89	206	423.5	1111.8
Netherlands	383	324	425	390	383	414	521	387	284	147	228	197	303	7081	26 863	2237.0	8765.7
Poland	36	50	68	80	64	59	48	80	46	93	61	80	68	75	333	10.3	389.7
Portugal	7	7	19	15	14	33	5	21	18	14	8	16	13	6	16	−53.8	23.1
Romania	25	15	23	20	28	24	21	18	21	20	17	22	28	62	8243	121.4	29 339.3
Russia	405 437	340 740	411 485	374 053	367 833	379 620	363 544	366 279	418 043	447 584	430 143	430 530	388 945	375 232	535 666	−3.5	37.7
Slovakia	317	232	337	327	535	431	197	317	181	231	255	243	428	1057	6090	147.0	1322.9
Spain	72	92	134	171	110	119	102	86	66	101	85	91	60	102	258	70.0	330.0
Switzerland	499	416	541	591	482	534	378	493	630	959	458	468	443	5226	34 783	1079.7	7751.7

## Discussion

The findings of this cross-sectional study are in line with recent reports from the media.^4^ The more pronounced increase in the sales of iodine-containing drugs in Eastern European countries (ie, Romania and Bulgaria) observed in this study may be related to the fact that these countries are geographically close to Russia and Ukraine and may, therefore, be more likely to suffer from collateral nuclear damage than other European countries. Interestingly, this is not the first time panic buying of iodine-containing drugs has occurred, and this behavior has also been reported after the Fukushima crisis.^5^ Panic buying of iodine-containing drugs since the beginning of the war is likely explained by the fear of the use of nuclear weapons and the fear of radiation leaks from nuclear sites.^2^ Despite international efforts, the nuclear threat is real, and this threat has been found to have adverse effects on mental health.^6^ Moreover, lack of awareness of international recommendations on iodine prophylaxis following nuclear accidents and inaccurate information from the media may potentiate the effects of the war between Russia and Ukraine on panic buying of iodine-containing drugs.

The major strengths of this study are the number of countries included in the analyses and the use of empirical data obtained every month. Two critical limitations are the absence of data on prescribed iodine-containing drugs and the lack of information on the sociodemographic and clinical characteristics of buyers of iodine-containing drugs.

## Conclusions

In conclusion, the war between Russia and Ukraine was associated with increased sales of over-the-counter iodine-containing drugs in 20 European countries in February and March 2022 compared with January 2022. On the basis of the findings of this study, there is an urgent need to better inform the general population about the international recommendations on iodine prophylaxis following exposure to radioiodine.

## Notes

Supplement. **eAppendix.** Supplemental Methods
eReferences
