# Supplementary material for: Sales of Iodine-Containing Drugs in Europe Following the Beginning of the War Between Russia and Ukraine
Source: JAMA Netw Open. 2022 Oct 27;5(10):e2240032. doi: 10.1001/jamanetworkopen.2022.40032 (PMC9614577; doi:10.1001/jamanetworkopen.2022.40032)
Supplement: Supplement. — eAppendix. Supplemental Methods eReferences [file jamanetwopen-e2240032-s001.pdf]

# Supplemental Online Content

Kostev K, Abeler S, Koyanagi A, Haro JM, Smith L, Jacob L. Sales of iodine-containing drugs in Europe following the beginning of the war between Russia and Ukraine. *JAMA Netw Open*. 2022;5(10):e2240032. doi:10.1001/jamanetworkopen.2022.40032

**eAppendix.** Supplemental Methods
**eReferences**

This supplemental material has been provided by the authors to give readers additional information about their work.

© 2022 Kostev K et al. *JAMA Network Open.*

**eAppendix. Supplemental Methods**

*Database*

The present pharmacoepidemiological study used data from the OTCims database (IQVIA). This database has already been used in prior scientific studies.[1,2] The OTCims database contains data on sales of over-the-counter drugs in America, Asia and Europe. For most European countries, data were collected for the first time 12 years ago. Data are sent to IQVIA every month, one to three months after the month of interest, depending on the country.

*Countries*

Twenty European countries were included in this study: Austria, Belgium, Bulgaria, Croatia, Czech Republic, Finland, France, Germany, Greece, Hungary, Italy, Latvia, Netherlands, Poland, Portugal, Romania, Russia, Slovakia, Spain, and Switzerland.

*Channels of distribution*

Channels of distribution were retail pharmacies, drugstores, supermarket in-store pharmacies and corners, supermarket aisles and shelves, online and mail orders, and other channels (e.g., hospital pharmacies and parapharmacies). Channels of distribution varied by country. Retail pharmacies were the main channel of distribution in all countries. Channels of distribution, sample size and coverage by country are displayed in **Table 1** in

© 2022 Kostev K et al. *JAMA Network Open.*

the main text. Retail pharmacies were the only channels of distribution of iodine-containing drugs in 35.0% of countries. The number of pharmacies was 59,287 in the overall sample, and ranged from 302 in Croatia to 14,000 in France. Finally, data were projected in 85.0% of countries.

*Type of data*

This study included sell-out data of drugs with iodine as the only active ingredient. Drugs with iodine as one of several active ingredients were excluded from the analyses. Sell-out data corresponded to sales from pharmacies to clients, and retainers were audited to obtain these data. Units corresponded to packages of iodine-containing drugs.

*Coverage*

Sales of packages of iodine-containing drugs were projected or not. When projected, sales corresponded to those obtained at the level of the country. When not projected, sales corresponded to those obtained at the level of the sample.

*Analyses*

The number of packages of iodine-containing drugs sold per month was analyzed in each country between January 2021 and March 2022. Differences in percentage were assessed between February 2022 and January 2022, and March 2022 and January 2022.

© 2022 Kostev K et al. *JAMA Network Open.*

**eReferences**

1. Tu CM. Use of Proprietary Names by Prescribers When Prescribing Over-the-Counter (OTC) Drug Products. *Ther Innov Regul Sci*. 2019;53(1):132-137. doi:10.1177/2168479018762376

2. Bousquet J, Schröder-Bernhardi D, Bachert C, et al. Heterogeneity of the pharmacologic treatment of allergic rhinitis in Europe based on MIDAS and OTCims platforms. *Clin Exp Allergy*. 2021;51(8):1033-1045. doi:10.1111/cea.13884

© 2022 Kostev K et al. *JAMA Network Open.*
